# Editorial: Computational Methods for Understanding Complexity: The Use of Formal Methods in Biology

**DOI:** 10.3389/fbioe.2016.00068

**Published:** 2016-08-22

**Authors:** David A. Rosenblueth

**Affiliations:** ^1^Instituto de Investigaciones en Matemáticas Aplicadas y en Sistemas, Universidad Nacional Autónoma de México, México D.F., Mexico

**Keywords:** Boolean networks, gene regulatory networks, biochemical networks, attractors of Boolean networks, logic programing, model checking, answer set programing, synthesis of biochemical models

**The Editorial on the Research Topic**

**Computational Methods for Understanding Complexity: The Use of Formal Methods in Biology**

The functional properties of living organisms have a complexity exceeding the human capacity for analysis. A basic conviction in computational biology is that it should be possible to develop computational tools allowing us to considerably increase our understanding of such functional properties.

Understanding a fragment of reality is closely related to having a model of such a fragment. Hence, model construction is a high priority on the agenda of computational biology. Once available, a model can then be analyzed with different techniques. These two processes, however, are often intertwined, as analysis can guide the construction of a model.

Among the *models* for biochemical and gene networks (de Jong, 2002; Fages and Soliman, 2008), ordinary differential equations are of prime importance. Stochastic models based on Gillespie’s method (identified with continuous-time Markov chains) represent perhaps a most concrete model. Discrete models (e.g., Petri nets) are prominent, as abstractions from stochastic techniques, where both the concentrations and time have been discretized. Finally, Boolean formalisms are abstractions of discrete models. Boolean models were initially studied with propositional logic (i.e., Boolean logic). Later, however, close connections with more expressive logics have been established, such as those underlying Logic Programing (Kowalski, 2014) and Model Checking (Clarke et al., 1999).

*Analysis techniques* vary in the direction of treatment of time. Simulators normally deal with time in a forward manner by reproducing in the model a single behavior among all possible behaviors from an initial state. Model checkers, by contrast, often proceed backwards by analyzing, in reverse, all possible behaviors ending in a given set of final states.

*Model-construction techniques*, in turn, range from those completely performed by a human being to those entirely mechanized.

Understanding a living system through a model could be a goal per se. The model of a system, nevertheless, can also be used for predicting or even controlling its behavior. Moreover, a model can be instrumental in the synthesis of a system itself.

The aim of the present research topic is to explore the application of formal methods for understanding biological systems. This research topic comprises nine articles. Five of them belong to the category Original Research, two are Reviews, one a Technology Report, and the last one an Opinion Article.

“Model Checking to Assess T-Helper Cell Plasticity,” by Abou-Jaoudé et al., is based on the discrete, asynchronous formalism developed by Thomas and D’Ari (1990). This work uses GINsim along with Model Checking for Action-Restricted Computation-Tree Logic (ARCTL). ARCTL is a generalization of ordinary Computation-Tree Logic (CTL) incorporating actions. This article extends a previously published work so as to cover several novel Th subtypes, and highlights the plasticity of Th cells depending on their microenvironment. The model has 101 variables (most of which, but not all, are Boolean) and 221 regulatory interactions.

“Approximating Attractors of Boolean Networks by Iterative CTL Model Checking,” by Klarner and Heike, is a contribution to the study of asynchronous Boolean networks. This article advocates a method for approximating asynchronous attractors by “minimal trap spaces” using Answer Set Programing (Eiter et al., 2009), a declarative problem-solving paradigm stemming from Logic Programing. Minimal trap spaces can be computed efficiently even for networks with hundreds of variables. To decide whether each minimal trap space contains exactly one attractor, and whether there are attractors outside them, the authors use CTL Model Checking.

“Systems Perturbation Analysis of a Large Scale Signal Transduction Model Reveals Potentially Influential Candidates for Cancer Therapeutics,” by Puniya et al., studies perturbations on a signal-transduction Boolean model having 132 variables and 557 interactions. Through simulations using the platform Cell Collective, this work suggests potential therapeutic targets.

“Learning Delayed Influences of Biological Systems,” by Ribeiro et al., is based on an extension of ordinary Boolean models with *delays* and employs Inductive Logic Programing to infer such models. Experimental data are a set of traces of observations, used in a bottom-up method that generates hypotheses. This process is illustrated in the yeast cell cycle system.

“Designing experiments to discriminate families of logic models,” by Videla et al., studies a method of synthesis of Boolean models employing Answer Set Programing. Through both prior knowledge and multiple-perturbation experiments thousands of logic models are retrieved. This is due to the incomplete and redundant nature of biological data. This work designs optimal experiments finding more specific logic models. The space of possible experiments is iteratively explored imposing constraints to minimize the number of input–output model behaviors at each step. The proposed method is applied to signaling pathways in human liver cells and phosphoproteomic data.

“Towards Synthesizing Executable Models in Biology,” by Fisher et al., discusses how Executable Biology can be aided by automatic synthesis of models. They exemplify this approach with several discrete models including a model of the *C. elegans* vulval precursor cells (VPC) system. The technique relies on the translation of the requirements from the model to logical constraints, which are supplied to a solver.

“A Survey about Methods Dedicated to Epistasis Detection,” by Niel et al., classifies epistasis-detection methods into those performing exhaustive search and those effecting non-exhaustive search. On the one hand, the exhaustive-search methods may or may not use filtering to reduce the size of the search space. On the other hand, the non-exhaustive-search methods use combinatorial optimization or machine-learning techniques.

“Systems Biology of Cancer: A Challenging Expedition for Clinical and Quantitative Biologists,” by Korsunsky et al., relates models with computer tools for computational biology. The models covered include Bayesian networks, Boolean networks, ordinary differential equations, and cellular automata. The computer tools encompass Model Checking and Sensitivity Analysis. Pancreatic cancer is used as an illustration.

“Normal vs. Malignant Hematopoiesis: The Complexity of Acute Leukemia through Systems Biology,” by Enciso et al., first observes that the relapse of acute leukemia could be explained as a selection eliminating highly proliferative cells due to chemotherapy, thus favoring slow-cycling cells. Hence, these authors advocate modeling both several hematopoietic populations and the interactions with non-hematopoietic neighboring cells.

We are in the course of learning what kind of model and what kind of analysis and model-building techniques to use for each particular problem. This research topic is a contribution to such exploration. There are articles employing well-established methods, adapting techniques to biology, and developing new approaches. We can also find discrete and Boolean models, and the use of both simulators and model checkers. At the same time, synthesis is exemplified both by manual and machine-learning methods. We believe that the articles in this research topic will stimulate new research.

## Author Contributions

DAR conceived the idea for this research topic, served as editor for the manuscripts, and wrote the editorial.

## Conflict of Interest Statement

The author declares that the research was conducted in the absence of any commercial or financial relationships that could be construed as a potential conflict of interest.
